# Endogenous c-Jun N-terminal kinase (JNK) activity marks the boundary between normal and malignant granulosa cells

**DOI:** 10.1038/s41419-018-0459-3

**Published:** 2018-03-16

**Authors:** Gamze Bildik, Nazli Akin, Filiz Senbabaoglu, Yashar Esmalian, Gizem Nur Sahin, Defne Urman, Sercin Karahuseyinoglu, Umit Ince, Erhan Palaoglu, Cagatay Taskiran, Macit Arvas, Yilmaz Guzel, Kayhan Yakin, Ozgur Oktem

**Affiliations:** 1 Graduate School of Health Sciences and School of Medicines, Koc University, Istanbul, Turkey; 2 Department of Histology and Embryology, School of Medicine, Koc University, Istanbul, Turkey; 3 Department of Pathology, School of Medicine, Acibadem University, Istanbul, Turkey; 4 American Hospital Clinical Biochemistry Laboratories, Istanbul, Turkey; 5 Department of Obstetrics and Gynecology, Gynecologic Oncology Division, School of Medicine, Koc University, Istanbul, Turkey; 6 Women’s Health Center, American Hospital, Istanbul, Turkey; 7 Department of Obstetrics and Gynecology, The Division of Reproductive Endocrinology and Infertility, Translational Research Laboratories, School of Medicine, Koc University, Istanbul, Turkey

## Abstract

Granulosa cell tumor of the ovary (GCT) is a very rare tumor, accounting for only 2% of all ovarian tumors. It originates from sex cords in the ovary and can be divided into adult (95%) and juvenile (5%) types based on histologic findings. To date, no clear etiologic process has been identified other than a missense point mutation in the *FOXL2* gene. Our previous works showed that c-Jun N-terminal kinase (JNK) pathway plays critical role in cell cycle progression and mitosis of normal and immortalized granulosa cells and follicle growth in rodent ovaries. These findings led us to investigate the role of JNK pathway in the granulosa cell tumor of the ovary. We used two different GCT cell lines (COV434 and KGN) and fresh GCT samples of adult and juvenile types obtained from the patients during surgery. We have discovered that endogenous kinase activity of JNK is markedly enhanced in the GCT samples and cell lines, whereas it was almost undetectable in mitotic non-malignant human granulosa cells. The inhibition of JNK pathway in GCT cell lines with two different pharmacologic inhibitors (SP600125 and AS601245) or siRNA resulted in a dose-dependent reduction in in vitro cell growth, increased apoptosis and diminished estradiol and AMH productions. JNK inhibition was also associated with a decrease in the number of cells positive for mitosis marker phospho-histone H3^Ser 10^ in the asynchronous cells; and diminished EdU uptake during S phase and cell cycle arrest at G2/M-phase transition in the synchronized cells. Ex vivo treatment of patient-derived GCT samples with JNK inhibitors for 24 h significantly decreased their in vitro growth and estradiol and AMH productions. Furthermore, in human GCT xenograft model, in vivo tumor growth was significantly reduced and plasma AMH levels were significantly decreased in SCID mice after administration of JNK inhibitors and siRNA. These findings suggest that targeting JNK pathway may provide therapeutic benefit in the treatment of granulosa cell tumors for which currently no curative therapy exists beyond surgery.

## Introduction

Granulosa cell tumor of the ovary (GCT) is a very rare tumor characterized by its tendency to recur years after the initial diagnosis. It accounts for approximately 2% of all ovarian tumors and can be divided into adult (95%) and juvenile (5%) types based on histologic findings^1,2^. To date, no clear etiologic process has been identified other than a somatic missense point mutation (C402→G; C134W) in the *FOXL2* gene that is positive in 97% of adult-type granulosa cell tumor and absent in its juvenile form^3^. Indeed, recent studies have revealed many genes and signaling pathways that are merged to FOXL2 and work as critical regulators of granulosa cell proliferation and function such transforming growth factor-β (TGF-β) signaling (GDF-9, follistatin, Smad3), GATA4 and aromatase^4–6^. Unlike the adult type, juvenile-type GCT (JGCT) is much rarer, does not harbor FOXL2 mutations and affects pre-pubertal girls and young women with a mean age of onset of around 8 years^7,8^. Its molecular mechanism is less known compared to adult type. One study detected in-frame tandem duplications within AKT1 as well as an array of point mutations altering highly conserved residues in a cohort of 16 JGCTs^9^. JGCTs exhibit reduced expression of FOXL2 compared to normal ovary^10^. Pre-ovulatory growth of the somatic cells of the ovary is induced by the follicle-stimulating hormone (FSH), and alterations in its signaling pathway have been suggested to play a role in tumorigenesis. Consistently, two activating mutations of the stimulatory α-subunit of a trimeric G protein (Gαs), located at position 201, have been identified in 30% of a JGCT cohort^11^. The majority of patients diagnosed with adult or juvenile GCT present with an early-stage disease, with a tumor limited to the ovary and have a good prognosis with a survival rate of >90% with surgery alone. However, patients with advanced-stage disease and widely spread tumors or recurrent cases have a very poor prognosis and are more difficult to treat. Anti-mullerian hormone (AMH) and estrogen are produced by hormonally active tumors and used as adjuvant hormone markers in the diagnosis and post-treatment follow-up of the patients. Because JGCTs are hormonally active, patients can be diagnosed with precocious pseudopuberty owing to increased estrogen secretion. Indeed, there are no other curative treatment forms other than surgery^9,12,13^.

Mitogen-activated protein kinases (MAPKs) are the members of a well-studied family of serine–threonine kinases that phosphorylate target proteins and play important regulatory roles in the cell.^14^ The c-Jun NH2-terminal kinases (JNKs), a member of MAPKs, are the master protein kinases that regulate many physiological processes, including inflammatory responses, cell proliferation, differentiation, survival and death^15,16^. Our previous work showed that FSH activates JNK pathway in rat granulosa cells, and when this pathway was blocked by pharmacological inhibitors, in vitro follicle growth is halted as a result of mitotic arrest in the granulosa cells surrounding the oocyte in the mouse model. We also found that JNK inhibition in spontaneously immortalized rat granulosa cells (SIGC) resulted in cell cycle arrest at G_2_/M transition^17,18^. This cell line represents an intermediate step in carcinogenesis because they grow indefinitely in culture but do not form clones in soft agar or tumors in nude mice^19^. Based on these findings we hypothesized that JNK pathway might have a role in GCT. For this purpose, we utilized fresh tumor samples of adult and juvenile types as well as two different GCT cell lines (COV434 and KGN) in comparison to immortalized mitotic non-luteinizing FSH-responsive human (HGrC1) and rat (SIGC) granulosa cell lines and primary human luteinized granulosa cells (HLGCs) obtained from In vitro fertilization (IVF) patients in this translational research study. Two different pharmacological JNK inhibitors (SP600125 and AS601245) and small interfering RNA (siRNA) technology were used to explore the role of JNK pathway in human GCT.

## Results

### Validation experiments

**Fig. 1:**
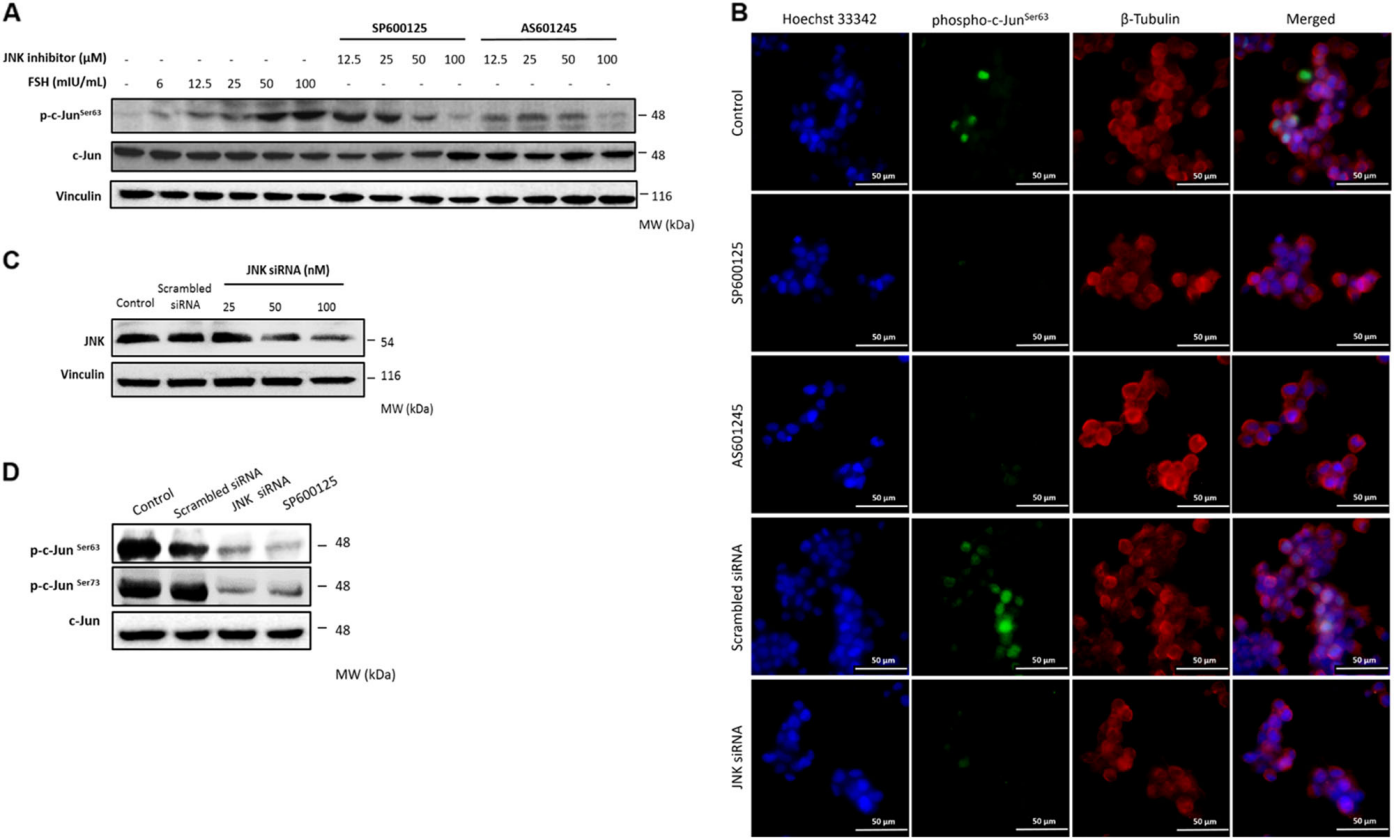
The results of validation experiments. FSH stimulation for 1 h activated JNK pathway in the GCT cell line COV434 as evidenced by a robust increase in phospho-c-Jun^Ser63^ level. Treatment with pharmacological JNK inhibitors SP600125 and AS601245 for 30 min at the indicated concentrations inhibited the phosphorylation of c-Jun in a dose-dependent manner in both western blot (**a**) and immunofluorescence staining (**b**). Knockdown efficiency of JNK siRNA was confirmed by decreased expression of total protein levels of JNK on western blot (**c**). Kinase assay confirmed that JNK activity was substantially inhibited with pharmacological inhibitors (50 μM) and siRNA (50 nM) (**d**). Scale bar: 50 µm

First, we conducted a series of validation experiments to test if our experimental model is suitable to study the role of JNK pathway in GCT cell lines and tumor samples. We previously determined in vitro growth characteristics of GCT cell lines COV434 and KGN, and mitotic non-malignant human (HGrC1) and rat (SIGC) granulosa cells^20^. In GCT cell line COV434, FSH stimulation at different concentrations increased the expression of phospho-c-Jun^Ser63^ in a dose-dependent fashion. Treatment with JNK inhibitors at 12.5, 25, 50 and 100 μmol/L concentrations 30 min before FSH abolished the expression of phospho-c-Jun^Ser63^ in a dose-dependent manner on western blotting (Fig. 1a) and immunofluorescence staining at low- and high-magnification images (Fig. 1b). Similarly, knockdown of JNK by siRNA at 25, 50 and 100 nM concentrations caused a dose-dependent decrease in the expression of protein level of total JNK (Fig. 1c). Inhibition of JNK pathway after treatment with pharmacological inhibitors and siRNA at the indicated concentrations was confirmed by measuring JNK activity with phosphorylation of c-Jun at Ser63 and 73 residues in GCT cell lines COV434 and KGN (Fig. 1d and supplementary figure-1).

### Inhibition of JNK pathway blocks proliferation and cell cycle progression of GCT cell lines

**Fig. 2:**
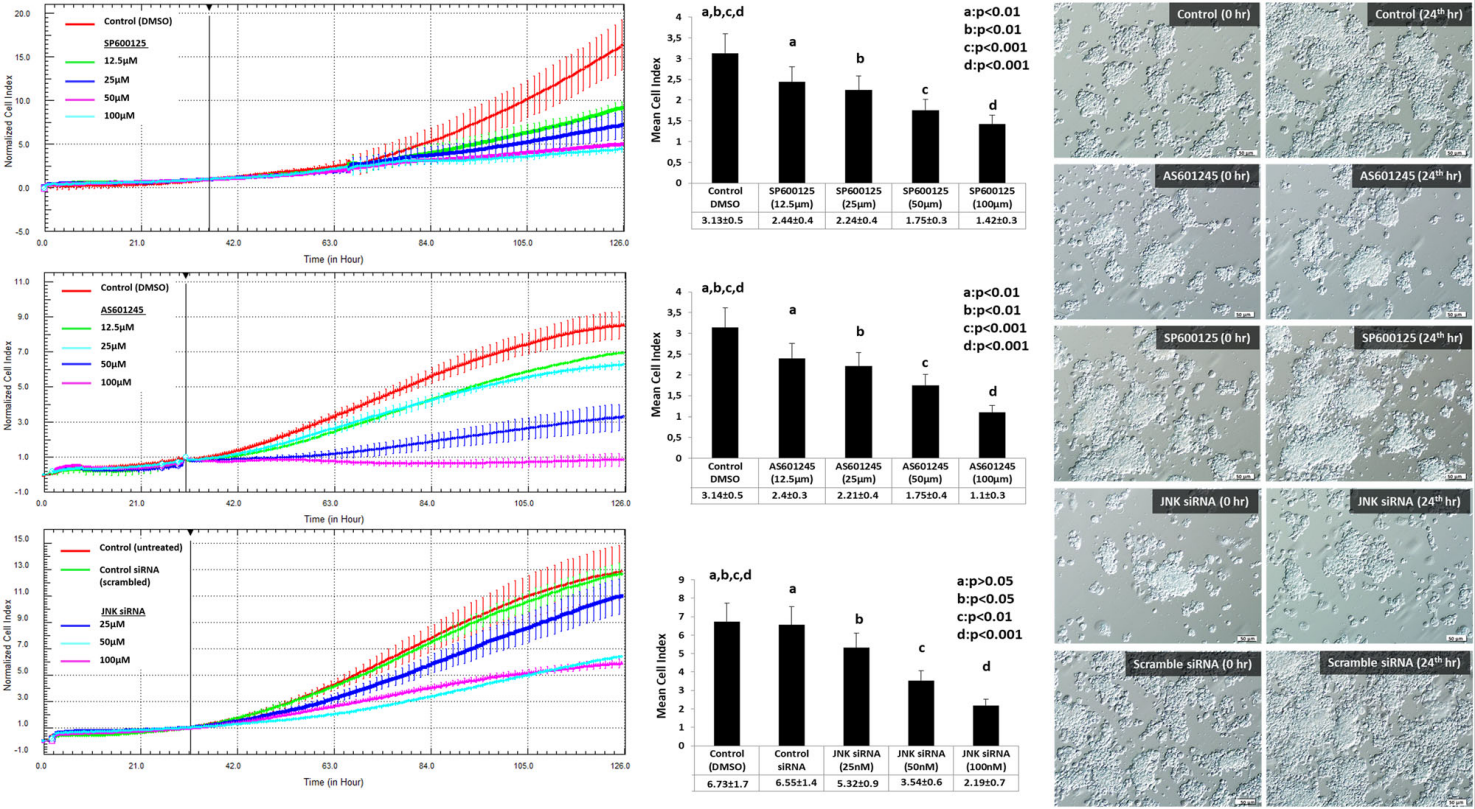
Interruption of JNK pathway via pharmacological inhibitors or siRNA blocks the proliferation of COV434 cells. Analysis of real-time growth curves of COV434 cells in the xCELLigence platform treated with JNK inhibitors SP600125 and AS601245, or transfected with JNK siRNA at the indicated concentrations revealed that there is dose-dependent reduction or arrest in in vitro proliferation of the cells after JNK inhibition (to the left of the panel). JNK inhibitors were administered when the cells reached log phase. For siRNA assay, transfection was done 48 h before re-plating the cells. Mean cell index calculated by the system as a measure of viable cell mass at the end of experiment period confirmed reduced proliferation of the cells after JNK inhibition either pharmacologically or via siRNA (shown as graph bars in the middle of the panel). Light microscopy images taken at time 0 and 24 h later for each group show arrested growth of the cells when JNK pathway was inhibited with SP60015-, AS601245- and JNK-specific siRNA in comparison to untreated control, vehicle (DMSO) and scrambled siRNA (to the right of the panel). Note that the cells were still at their seeding confluency 24 h after JNK inhibition (scale bar: 50 µm)

**Fig. 3:**
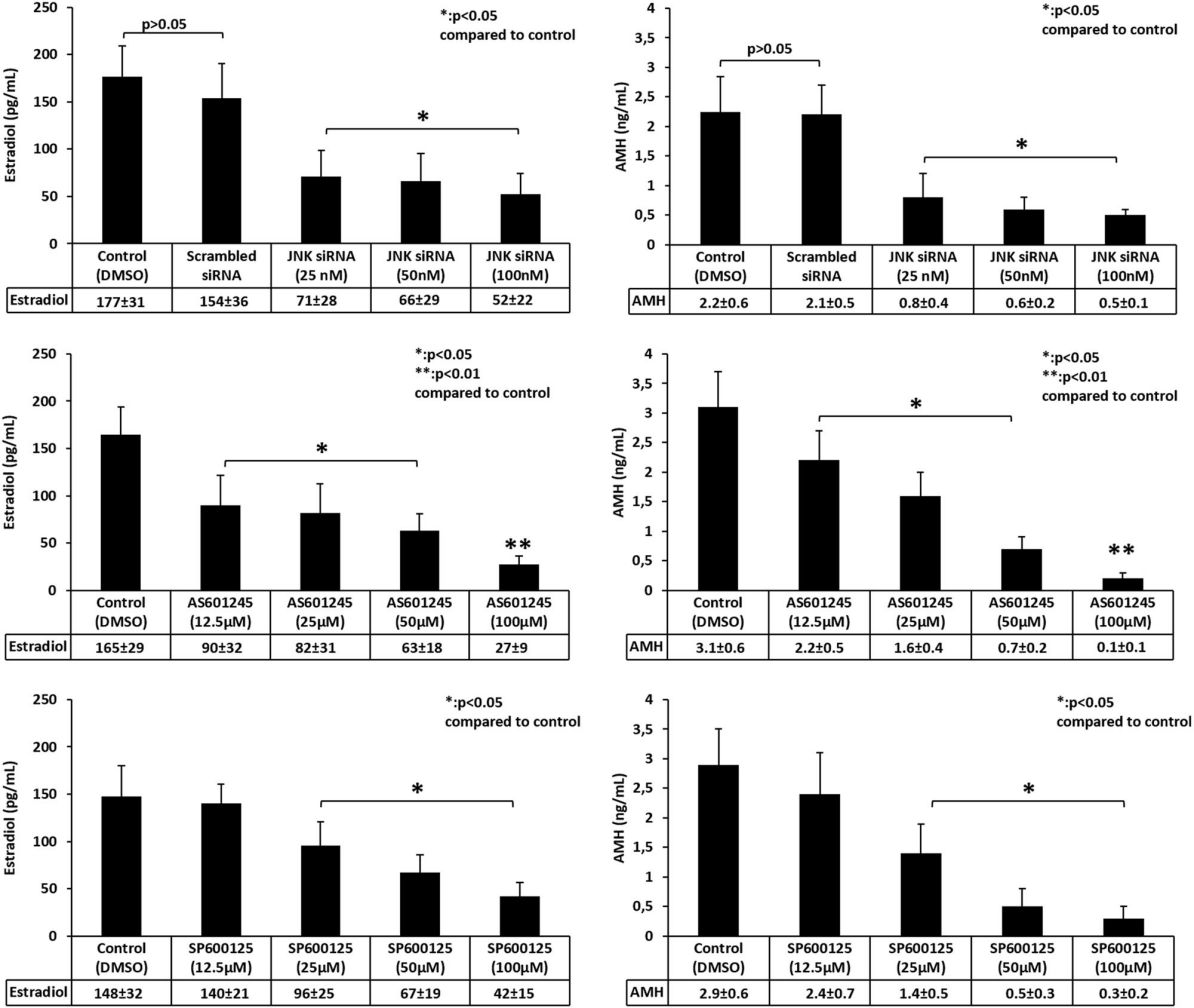
In vitro estradiol and anti-mullerian hormone (AMH) production of the COV434 cells before and after JNK inhibition with pharmacological inhibitors and siRNA. Measurement of estradiol and AMH productions in the spent culture media of the COV434 cells treated with JNK inhibitors or transfected with siRNA for 24 h showed that JNK inhibition with either pharmacological inhibitors or siRNA was associated with reduced in vitro hormone production of the cells in a dose-dependent fashion

The interruption of JNK pathway via either pharmacologic inhibitors (SP600125 and AS601245) or siRNA at the indicated concentrations resulted in a dose-dependent decrease or arrest in the proliferation of both COV434 and KGN cells as evidenced by real-time and quantitative assessment of growth curves of the cells and their mean cell indices in the xCELLigence system (Fig. 2 and supplementary figure-2). In line with these results, in vitro estradiol and AMH production of the cells were significantly decreased when JNK pathway was inhibited either pharmacologically or via siRNA (Fig. 3).

**Fig. 4:**
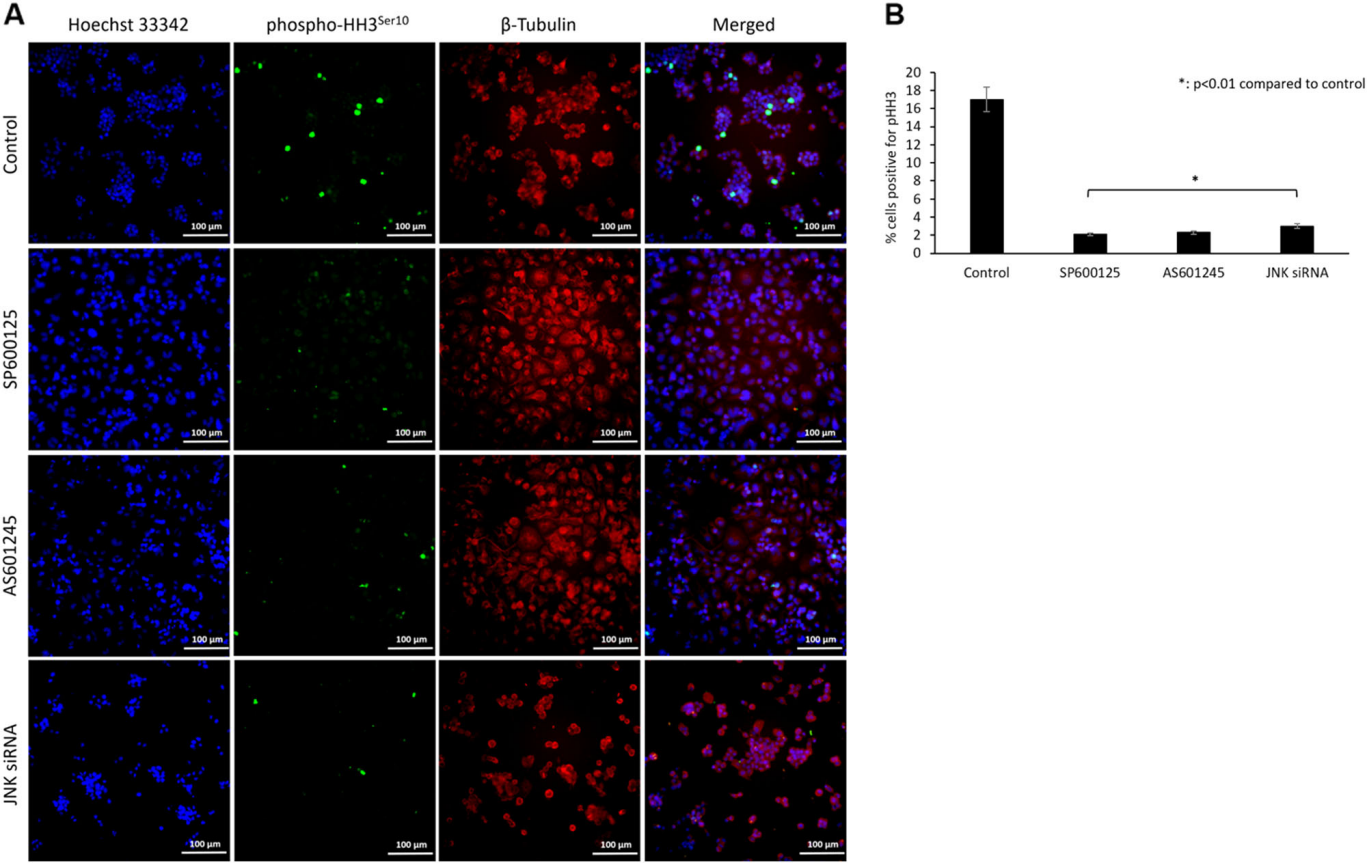
JNK inhibition is associated with reduced expression of mitosis marker phospho-histone H3^Ser10^ in COV434 cells. JNK inhibition in asynchronous COV434 cells is associated with decreased mitotic activity as evidenced by reduced expression of mitosis marker phospho-histone H3^Ser10^ on immunofluorescence analysis (**a**). While 17% of the control cells were stained positive for pHH3^Ser10^, this rate was significantly reduced when JNK was inhibited either pharmacologically (2%) or via siRNA (3%) (**b**). Scale bar: 100 µm

In asynchronous COV434 cells, reduced growth was also associated with a significant decrease in the number of cells stained positive for mitosis marker phospho-histone H3^Ser10^ after JNK inhibition with pharmacological inhibitors (2%) and siRNA (3%) compared to control cells (17%, *p* < 0.01) on immunofluorescence staining (Fig. 4a, b). A similar reduction in the percentage of the cells positive for phospho-histone H3^Ser10^ was also observed in asynchronous KGN cell line after JNK inhibition with pharmacological inhibitors and siRNA (Supplementary Figure-3B and 3B).

**Fig. 5:**
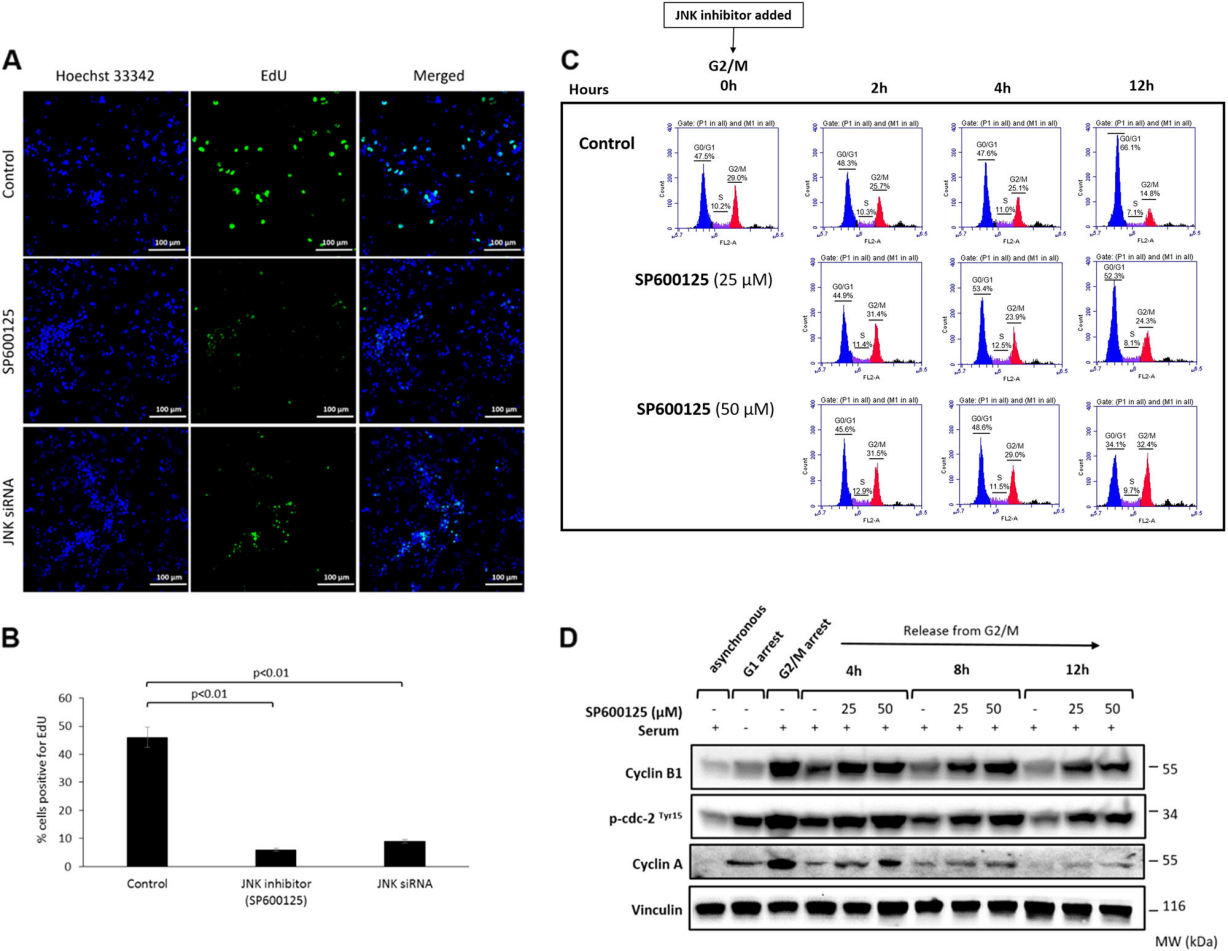
The effect of JNK inhibition on cell cycle progression of COV434 cells. Cell cycle progression at G1/S and G2/M before and after JNK inhibition was analyzed by EdU uptake and flow cytometry with the expression of the cyclins on western blot, respectively. JNK inhibition at G1/S was associated with reduced EdU uptake (**a**, **b**). The cells accumulated and failed to exit mitosis when JNK was inhibited at G2/M transition as quantitatively shown by the fraction of the cells by flow cytometric analysis (**c**) and the absence of de-phosphorylation of p-cdc2^Thy15^ and degradation of cyclins A and B on western blot analysis (**d**). Scale bar: 100 µm

We further explored the role of JNK pathway in GCT by analyzing cell cycle progression at G1/S and G2/M transitions after JNK inhibition in GCT cell lines. The 5-ethynyl-2'-deoxyuridine (EdU) uptake of the COV434 cells synchronized at G1/S was significantly decreased after JNK inhibition with pharmacological inhibitors (6%) or siRNA (9%) in comparison to controls (47%, *p* < 0.01, Fig. 5a, b). Similar results were obtained in KGN cell line (Supplementary Figure-4). JNK inhibition at G2/M transition resulted in failure of the cells to exit mitosis and caused their accumulation at G2/M as evidenced by flow cytometric analysis and absence of de-phosphorylation of p-cdc-2^Tyr15^ and degradation of cyclins A and B1 on western blotting (Fig. 5c, d). High-magnification images for the immunofluorescence staining experiments for phospho-c-Jun and phospho-histone-H3 can be found in the supplementary figure. 5.

### Endogenous kinase activity of JNK is higher in neoplastic granulosa cells compared to primary and immortalized non-neoplastic granulosa cells

**Fig. 6:**
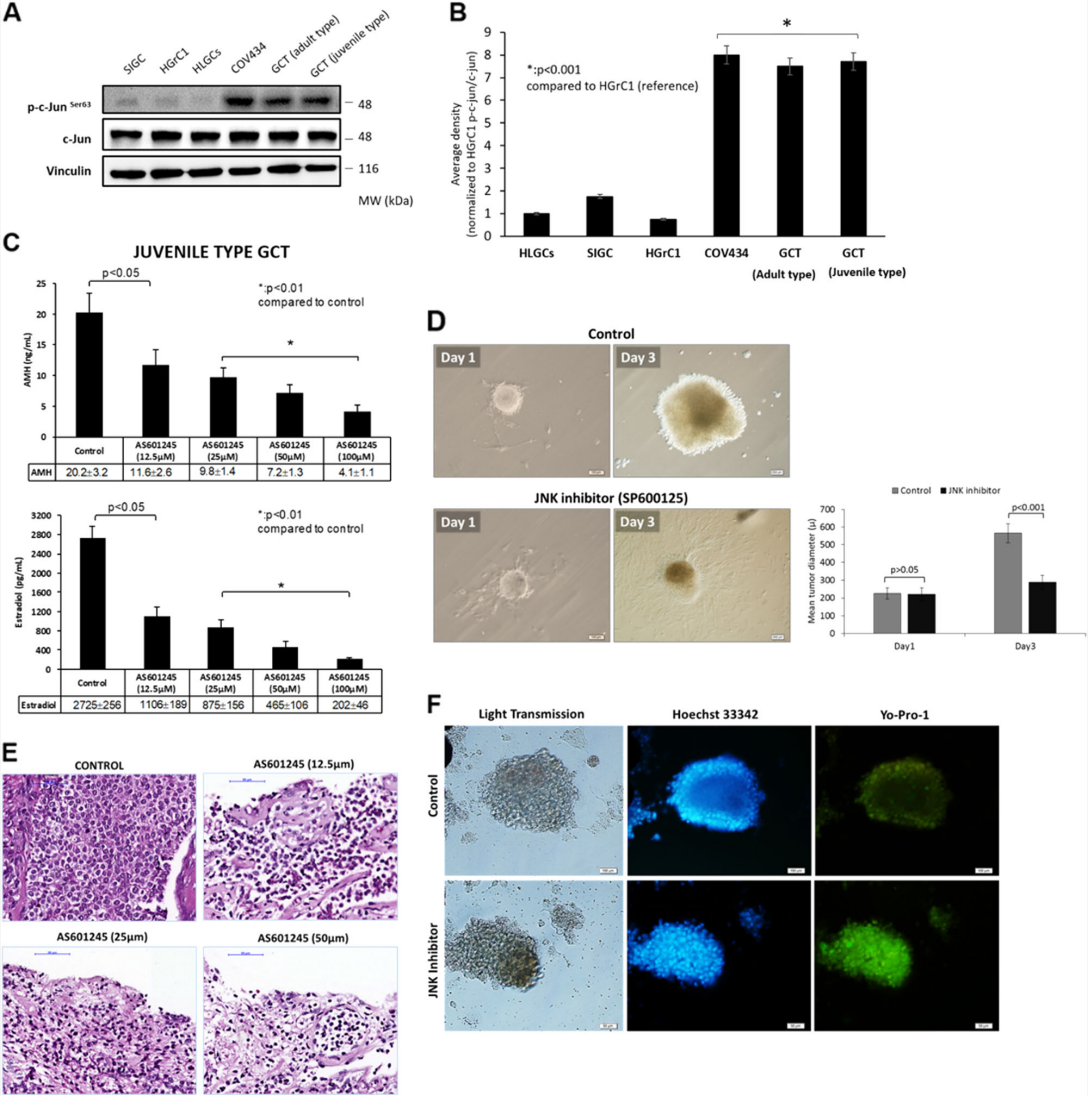
Comparison of JNK activity among normal and neoplastic granulosa cells. There is a marked increase in the endogenous kinase activity of JNK in patient-derived GCT samples and COV434 and KGN cell lines in comparison to other types of normal non-neoplastic granulosa cells of human and rat origin. HLGC human luteal granulosa cells, HGrC1 human mitotic non-luteinized granulosa cells, SIGC spontaneously immortalized rat granulosa cells (**a**,** b**). Ex vivo treatment of the GCT samples with JNK inhibitors caused a dose-dependent decrease in their estradiol and AMH productions in vitro (**c**) and in tumor growth (**d**). JNK inhibition was also associated with decreased viability of the samples on histological examination and immunofluorescence analysis (**e**, **f**). Scale bar: 100 µm for (**d**, **f**), and 50 µm for (**e**)

Robust anti-proliferative effect of JNK inhibition on the GCT cell lines COV434 and KGN led us to measure JNK activity in GCT cell line and fresh tumor samples in comparison to normal granulosa cells. We found that endogenous kinase activity of JNK is significantly increased in the tumor samples of both adult- and juvenile-type GCT as well as GCT cell lines COV434 and KGN in comparison to other types of non-neoplastic granulosa cells (HGrC1, HLGC and SIGCs) (Fig. 6a, b and Supplementary Figure-6). Ex vivo treatment of fresh GCT samples of both adult and juvenile types with JNK inhibitors in culture resulted in a significant decrease in their in vitro growth and estradiol and AMH productions compared to control samples (Fig. 6c, d and Supplementary Figure-7). Histological examination and cell death assay with intra-vital fluorescein carbocyanine uptake confirmed decreased viability and increased cell death in the samples treated with JNK inhibitors (Fig. 6e, f).

### JNK inhibition halts in vivo growth of GCT in mice

**Fig. 7:**
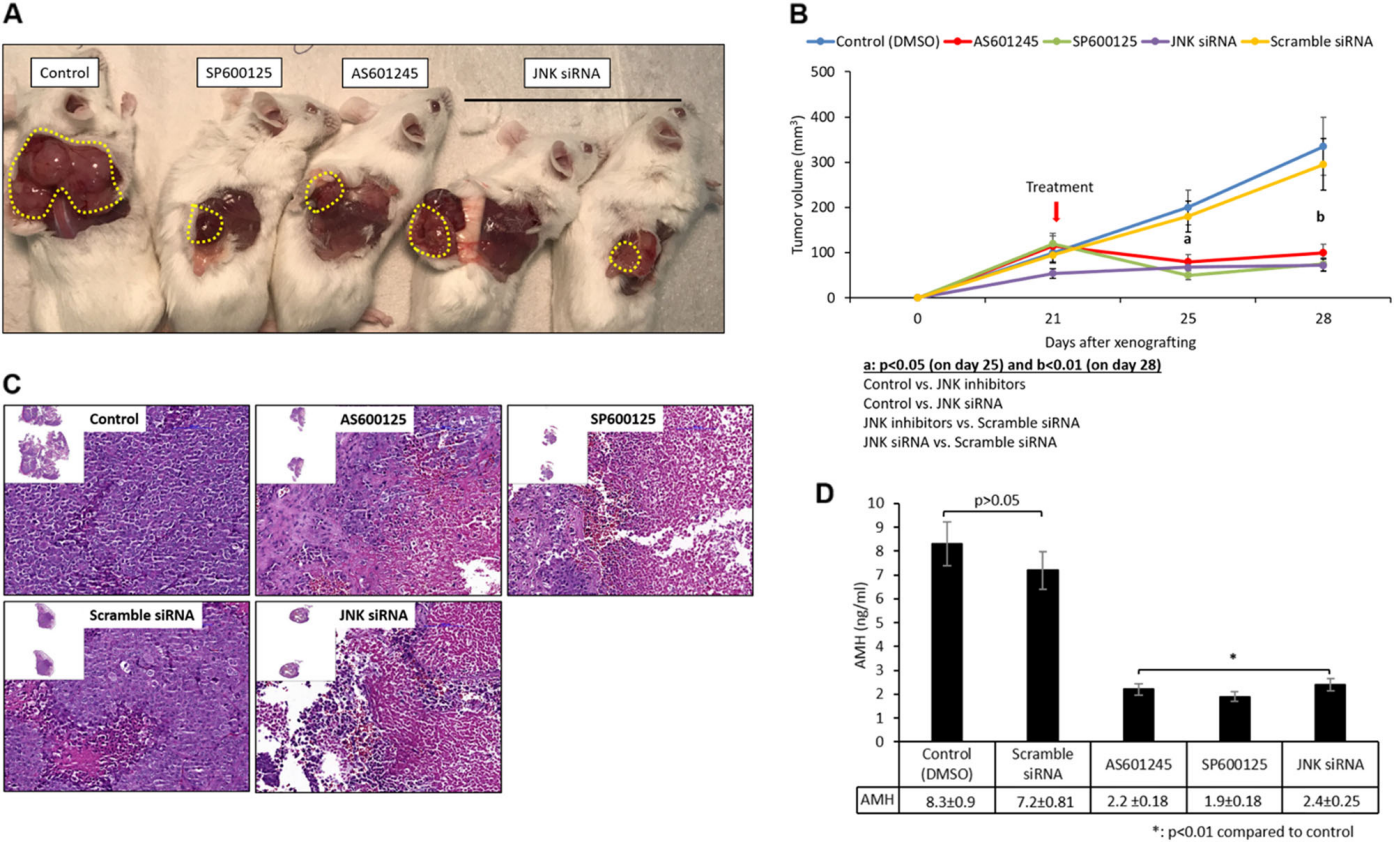
Human GCT xenograft experiment. Inhibition of JNK pathway either pharmacologically with its specific inhibitors (SP600125 and AS601245) or via siRNA resulted in a significant decrease in in vivo growth of GCT cell line (COV434) when xenografted subcutaneously into SCID mice (**a**, **b**). In line with these results, histopathological examination showed that there was a marked decrease in cellularity in the tumors with a concomitant decrease in the serum AMH level in the animals after JNK inhibitor treatment was given (**c**,** d**)

So far, our results showed that the inhibition of JNK pathway via either pharmacologically or RNA interference technology significantly blocked in vitro proliferation of tumor cells, caused cell cycle arrest and decreased in vitro growth of tumor explants. To further substantiate the significance of our findings, we have generated a human GCT xenograft model to analyze the effects of JNK inhibition on in vivo growth of GCT. As shown in the Fig. 7a–d, in vivo growth of the GCT (COV434) was significantly halted and serum AMH levels were significantly decreased after systemic and intra-tumoral administration of pharmacological JNK inhibitors and JNK siRNA, respectively, in these animals. While control and scrambled siRNA-injected tumors continue to grow in vivo on day 28 post xenografting, such a growth was not observed in those treated with JNK inhibitors or JNK siRNA.

## Discussion

We have obtained several important findings in this study. First, endogenous kinase activity of JNK in GCT samples of adult and juvenile types and cell lines is higher than non-neoplastic granulosa cells. Second, interruption of JNK signaling in GCT cell lines via pharmacologic inhibitors and siRNA inhibited their mitotic proliferation, reduced EdU uptake during S phase and blocked cell cycle progression at G2/M transition. Third, ex vivo treatment of fresh GCT of tumor samples of both adult and juvenile types with JNK inhibitors was associated with a marked reduction in in vitro growth, hormone productions and viability of these tumors. Fourth, in vivo growth of the GCT cell line (COV434) when xenografted into SCID (severe combined immune deficient) mice was significantly halted and serum AMH levels were significantly decreased after systemic and intra-tumoral administration of pharmacological JNK inhibitors and JNK siRNA, respectively, in these animals. Taken together, these findings suggest that JNK signaling pathway may have a role in the underlying pathogenesis of this rare type of ovarian tumor in humans.

It is well documented that JNK pathway and its downstream effector c-Jun plays crucial role in cell cycle regulation and mitosis of many neoplastic and non-neoplastic cells^15^. Therefore, higher endogenous kinase activity of JNK detected in GCT tumor samples and cell lines might not be surprising. However, absence of such an increase in JNK activity in immortalized mitotically active and rapidly proliferating normal granulosa cells of human (HGrC1) and rat (SIGC) origin suggests that enhanced kinase activity of JNK cannot be solely explained by the presence of mitotic activity in GCT samples and cell lines.

Our study is not the first one that documents the important role of JNK signaling pathway in proliferation and cell cycle progression of malignant tumors as this issue was previously extensively studied in malignant tumors such as the ones showing inhibition of JNK reduces G2/M transit independent of p53, leading to endoreduplication, decreased proliferation and apoptosis in the cell lines of breast, lung, thyroid and cervical cancer^21–24^. However, our findings could be still important given that very limited data are available regarding signaling pathways that act as gate-keeper of the proliferation and apoptosis of granulosa cell tumor. To date, no clear etiologic process has been identified other than a somatic missense point mutation (C402→G; C134W) in the *FOXL2* gene that is positive in 97% of adult-type granulosa cell tumor and absent in its juvenile form^3^. Indeed, recent studies have revealed many genes and signaling pathways that are merged to FOXL2 and work as critical regulators of granulosa cell proliferation and function such as TGF-β signaling (GDF-9, follistatin, Smad3), GATA4 and aromatase^4–6^. Unlike the adult type, JGCT is much rarer, does not harbor FOXL2 mutations and affects pre-pubertal girls and young women with a mean age of onset of around 8 years^7,8^. Its molecular mechanism is less known compared to adult type.

Quiescent primordial follicles are composed of a single layer of flattened granulosa cells surrounding the oocyte. Activation of primordial follicles and their transformation into primary stage and beyond is termed initiation of follicle growth and is characterized by squamous to cuboidal transformation of granulosa cells and their further mitotic proliferation. Current evidence suggests that there is a delicate balance of activating and suppressing signaling pathways and the genes that tightly control the process of follicle activation^25^. It is noteworthy that the genes that prevent premature activation of primordial follicles may also cause the formation of granulosa cell tumors when they are mutated. *FOXL2* is the first, and probably the most striking, example of how a single gene is a critical regulator of ovarian function, morphology and neoplasia formation in humans. FOXL2 is a winged-helix/forkhead domain transcription factor. Germ-line knockout of *Foxl2* is associated with a failure to assemble primordial follicles^26^, while mutations to *Foxl2* cause blepharophimosis/ptosis/epicanthus inversus syndrome (BPES) type I, an autosomal-dominant disorder associated with premature ovarian failure^27^. The relationship between *FOXL2* mutations and premature ovarian failure in women with BPES had not been known until 2004, when a transgenic mouse study provided the molecular link^28^. Five years later, a group of investigators identified a missense point mutation (402C>G [C134W]) causing a reduction in *FOXL2* gene expression in a granulosa cell tumor of the ovary^3^. In the same year, Kobel et al.^29^ identified the same mutation in nearly 90% of the cases of adult-type granulosa cell tumors. Subsequent studies revealed that the *FOXL2* gene is involved in many vital processes of granulosa cells including homeostasis, modulation of the expression of key genes in the differentiation and steroidogenesis, regulation of cell survival and proliferation and inhibition of cell cycle progression. Furthermore, whereas wild-type *FOXL2* can induce apoptotic death of granulosa cells and halt cell cycle progression, the C134W mutant cannot, thus providing a survival advantage to malignant granulosa cells^6,30–33^. By the same token, if the *FOXL2* gene functions to contribute to follicle dormancy and its mutations can cause premature activation of primordial follicles and/or favor the formation of granulosa cell tumors, can other similar factors or the genes do the same in the granulosa cells? Emerging data from animal studies suggest that Forkhead box O3 (FOXO3), phosphatase and tensin homolog (PTEN) and mammalian target of rapamycin (mTOR) pathways converge on the same follicle dormancy and granulosa cell tumor formation phenotypes. For instance, when *Foxo1* and *Foxo3* genes were selectively inactivated follicle development is impaired and granulosa cell tumor formation is increased in a mouse model^34^. Moreover, deletion of *Pten* in this mouse strain enhances the penetrance and accelerates the onset of granulosa cell tumor formation^34^. Other studies showed as well that targeted deletion of *Pten* alone or in combination with an activating phosphatidylinositol-3-kinase (PI3K) mutation can give rise to granulosa cell tumors in mice^35,36^. However, there is little evidence in humans that links these genes to premature activation of primordial follicles and the formation of granulosa cell tumors. Only two studies in the literature addressed this question at all. The first study by Bittinger et al.^37^ found that the granulosa cell tumor lines, COV434 and KGN, had no mutations within the *PTEN* gene nor increased expression *PIK3CA* and *PIK3R1*, which encode the catalytic and regulatory subunits, respectively, of PI3K. However, the relevance of these findings to primary human granulosa cell tumor is not clear as the authors could not confirm their results in the surgical GCT samples removed from patients. The second study by Rico et al.^38^ found an increase in the protein abundance of mTOR and its downstream effectors—RPS6KB1 (ribosomal protein S6 kinase B1), RPS6 (ribosomal protein S6), eIF4B (eukaryotic translation initiation factor 4B) and PPARG (peroxisome proliferator-activated receptor gamma)—in three human granulosa cell tumors samples relative to normal granulosa cells. In the latter study, the investigators also generated a transgenic mouse model in which mTOR, RPS6KB1, eIF4B and PPARG are up-regulated in a manner similar to that found in human granulosa cell tumors. Daily treatment of these animals with the mTOR-specific inhibitor Everolimus for 3 weeks significantly decreased the volume and growth rate of the tumors, and increased the survival of the animals^38^.

### Conclusion

Our findings warrant further investigations since targeting JNK pathway may provide therapeutic benefit in the treatment of granulosa cell tumors for which currently no curative therapy exists beyond surgery.

## Materials and methods

### Patients

GCT samples of adult (*n* = 2) and juvenile types (*n* = 2) were obtained from 4 patients. Adult and juvenile types of GCT samples were diagnosed and confirmed by two expert pathologists in gynecologic tumors. Non-mitotic HLGCs were isolated from follicular fluid of 10 IVF patients after oocyte retrieval procedure. Informed consents were obtained from all patients and the study was approved by the institutional review board of Koc University.

### Chemicals and reagents

All cell culture materials were obtained from Gibco Inc. SP600125 and AS601245, pharmacological inhibitors of JNK, were purchased from Calbiochem. Control (#6568) and SAPK/JNK (#6232) siRNA, SAPK/JNK Kinase Assay Kit (#8794, nonradioactive), Hoechst 33342 (#4082), Anti-c-Jun (#9165), Anti-Phospho-c-Jun^Ser63^ (#12598), Anti-Phospho-c-Jun^Ser73^ (#3270 S), Anti SAPK/JNK (#9252), Anti-Phospho-SAPK/JNK^Thr183/Tyr185^ (#9251), Anti-Cyclin A (4656), Anti-Cyclin B1 (#12231) and Anti-Phospho-cdc-2^Tyr15^ (#4539) antibodies were obtained from Cell Signaling. All western blotting buffers and reagents were purchased from Bio-Rad. Anti-phospho-Histone H3^Ser10^ (06-570) antibody was obtained from Upstate. Anti-Vinculin Antibody (V9264) was purchased from Sigma-Aldrich. TransIT-X2® Dynamic Delivery System (MIR 6000) was purchased from Mirus Bio LLC. YO-PRO®-1 Iodide (491/509) was obtained from Life Technologies. Texas Red™-X Phalloidin and Click-iT™ EdU Alexa Fluor™ 488 Imaging Kit (C10337) were obtained from Thermo Fisher Scientific. Matrigel Basement Membrane Matrix (356230) was from BD Biosciences. Super Block reagent (#AAA125) was purchased from ScyTek Laboratories.

### Cell lines

COV434 is a commercially available cell line derived from a granulosa cell tumor. The biological characteristics of this cell line include the production of 17β-estradiol in response to FSH, the absence of LH receptor, no lutenization capability and the presence of specific molecular markers of apoptosis enabling the induction of follicular atresia^39^.

KGN is also a steroidogenic human ovarian granulosa-like tumor cell line derived from a patient with invasive ovarian granulosa cell carcinoma. KGN cells are able to secrete pregnenolone and progesterone with little or no secretion of 17-α-hydroxylated steroids, androstenedione or estradiol. Fas-mediated apoptosis was also demonstrated in this cell line, which mimics the physiological regulation of apoptosis in human granulosa cells^40^.

HGrC1 is a human non-luteinized granulosa cell line expressing enzymes related to steroidogenesis, such as steroidogenic acute regulatory protein, aromatase and gonadotropin receptors. These cells are not capable of undergoing luteinization, resembling the characteristics of granulosa cells belonging to follicles in the early stage. HGrC1 might also be capable of displaying the growth transition from a gonadotropin-independent status to gonadotropin-dependent one^41^.

Non-mitotic luteinized human granulosa cells (HLGCs) were recovered from follicular fluid during oocyte retrieval procedure in 10 IVF patients. These cells are highly specialized primary luteinized granulosa cells, and they do not proliferate either spontaneously;or after stimulation with a mitogenic agent. They produce large amounts of progesterone and estradiol hormones in vitro^20^. The aspirates of follicular fluids were spun down at 500 × *g* for 10 min and then the cells were recovered and plated.

Spontaneously immortalized rat granulosa cells (SIGC) have an epithelial morphology, express gonadotropin receptors and grow in culture indefinitely. It is a spontaneously immortalized clonal granulosa cell line derived from primary rat ovarian granulosa cells and represents an intermediate step in carcinogenesis because they grow indefinitely in culture but do not form clones in soft agar or tumors in nude mice.^19^ They respond to FSH stimulation with enhanced growth but without undergoing luteinization^19^, resembling the growth characteristics of the proliferating granulosa cells of preantral and early antral follicles as well^42,43^.

### Cell culture

All granulosa cell lines and HLGCs were maintained at 37 °C with 5% CO_2,_ in Dulbecco's modified Eagle's medium (DMEM)/F12 supplemented with 10% (v/v) fetal bovine serum and 1% (v/v) penicillin–streptomycin *Amphotericin* B Solution, hereafter referred as complete media. The cells were routinely harvested by trypsinization with 0.25% trypsin–EDTA, counted using a hemocytometer and 0.4% Trypan blue.

### Human GCT tissue culture

Surgical GCT samples of adult and juvenile types were either freshly lysed for measurement of JNK activity or prepared for in vitro culture. Tumor samples were finely minced and dispersed to cells by treatment with 0.25% collagenase at 37 °C for 1 h, plated into 6-well plates and incubated overnight. The next day, experiment groups were treated with fresh complete media containing JNK inhibitors for 72 h, while the media of control group contained dimethyl sulfoxide (DMSO). Cultured groups were then used for hematoxylin and eosin staining and YO-PRO-1 assay to compare cell viability. Estradiol and AMH levels were measured in culture medium.

### Human GCT xenograft model in SCID mice

GCT cell line COV434 was re-suspended in growth factor reduced Matrigel^®^ diluted with DMEM/F12 and xenografted subcutaneously to the right flank region of the SCID mice (*n* = 4 per group) as 1 million cells/300 µl suspension with an insulin syringe. Tumor volume was monitored every week. Once palpable tumor growth was achieved at approximately 3 weeks post transplantation, JNK inhibitors SP600125 (10 mg/kg) and AS601245 (10 mg/kg) were administered systemically in 100 μL of DMSO via intraperitoneal route as a single injection (day 21). Control animals received DMSO only. For siRNA experiments, in vitro siRNA transfection experiments were carried out first and then transfected cells were xenografted in the same manner 48 h later as 1 million cells/300 µl suspension with an insulin syringe. JNK or scrambled siRNA were given as a single intra-tumoral injection on day 21. Tumor growth was followed for another week post injection on day 28, and the animals were killed, tumors were removed and intra-cardiac blood samples were obtained immediately post-mortem for AMH measurement.

### SAPK/JNK kinase assay

Intracellular JNK activity was measured by non-radioactive assay kit as instructed by the manufacturer. Briefly, the cells and tissues were harvested under non-denaturating conditions, lysed on ice and centrifuged at 14,000 × *g* for 10 min at 4 °C. Then, 200 µl of cell/tissue lysates was incubated with 20 µl phospho-JNK rabbit monoclonal antibody linked to agarose beads to precipitate JNK enzyme. After addition of necessary buffers, c-Jun substrate and adenosine triphosphate, reaction mixture was incubated for 30 min at 30 °C, optimal reaction condition that allows c-Jun substrate to be phosphorylated by precipitated JNK. The reaction was stopped by adding 4× SDS sample buffer and the samples were loaded onto 10% polyacrylamide gel. Then, proteins were transferred to polyvinylidene difluoride (PVDF) membrane by electroblotting and monoclonal antibodies were used to measure JNK-induced phosphorylation of c-Jun substrate at Ser63 and Ser73 residues.

### Western blotting

For western blot analysis, the cells were harvested, washed with ice-cold phosphate-buffered saline (PBS) and then lysed on ice RIPA buffer with protease and phosphatase inhibitors. Equal amount of protein per lane was separated by electrophoresis using 10% Tris-Glycine polyacrylamide gels. Proteins were transferred to PVDF membranes and blocked for 1 h with 5% non-fat dry milk at room temperature. Overnight incubation at 4 °C with primary antibody was performed in recommended dilutions. Anti-Vinculin antibody at a dilution of 1:10,000 is used as a loading control.

### Transfection with siRNA

COV434 and KGN cells were seeded in 6-well plates and cultured until 70% confluency was obtained. TransIT-X2 Dynamic Delivery System was used to perform siRNA transfection at concentrations of 12.5, 25, 50 and 100 nM. The cells were incubated for 48 h post transfection, then harvested to determine the transfection efficiency by JNK expression on western blot.

### Real-time monitoring of cell proliferation via xCELLigence System

The xCELLigence system uses interdigitated gold microelectrodes containing plates (E-plate) to non-invasively monitor the viability of cells using electrical impedance as the readout and generates real-time curves of cell viability and proliferation. The electronic readout of cell–sensor impedance is displayed in real-time as cell index (CI), a value directly influenced by cell attachment, spreading and/or cell proliferation. The cells were seeded in 16-well E-plate at the density of 10,000 cells per well in a final volume of 200 μL and incubated at 37 °C with 5% CO_2_ and continuously monitored on the RTCA system at 30 min time intervals. When the cells reached the log growth phase, they were treated with 12.5, 25, 50 and 100 μM concentrations of both SP600125 and AS601245. The effects of these inhibitors on viability and proliferation of COV434 were monitored up to 120 h. The results were expressed by normalized CI which are derived from the ratio of CIs before and after the addition of the compounds. The normalization of CI arbitrarily sets CI to 1 at the indicated time points. Recording of CI and normalization CI was performed using the RTCA Software 1.2.

### Immunofluorescent staining

For immunofluorescence studies, cells grown on coverslips were fixed with 4% paraformaldehyde for 20 min at room temperature, permeabilized with 0.1% Triton X-100 and then treated with superblock for 10 min. After rinsing with DPBS-T (0.1% Tween-20 in Dulbecco’s PBS), they were incubated with pHH3 antibody in superblock at 1:50 dilution overnight at 4 °C. The cells were washed with DPBS-T and incubated with fluorochrome-conjugated secondary antibody diluted in PBS for 90 min at 37 °C. This step was followed by rinsing the coverslips and adding Hoechst 33342 for DNA staining. The images were taken under appropriate channels using Immunofluorescence (IF) microscope. The percentage of the pHH3-positive cells was calculated after counting 500 cells at four different high-magnification areas.

### EdU assay

Cells plated on coverslips were synchronized with serum starvation for 24 h, then treated with complete media containing 2 μg/mL aphidicolin for 18 h. Then, the cells synchronized at G1/S were treated with JNK inhibitor SP600125 for 1 h while control group was incubated in complete media. After treatments, to detect newly synthesized DNA, the cells were incubated with of 10 μM EdU in culture medium for 2 h and fixed for imaging.

### Assessment of cell viability with YO-PRO®-1 staining

During apoptosis, the cytoplasmic membrane becomes slightly permeant to certain dyes, such as the green fluorescent YO-PRO®-1. After staining with YO-PRO®-1, apoptotic cells show green fluorescence while live cells show little or no fluorescence. The cells were treated with SP600125 and AS601245 for 24 h, then stained with YO-PRO®-1 (1 µM) and incubated for 20 min at 37 °C. The images were taken under appropriate channels using IF microscope. In all, 500 cells were counted at four different high-magnification areas and the percentage of the YO- PRO®-1-positive cells was calculated.

### Cell synchronization and flow cytometry analysis

For synchronization at G1/S, cells were serum starved for 24 h and then treated with complete media containing 2 μg/mL aphidicolin for 18 h. Then, the cells synchronized at G1/S by aphidicolin were incubated in complete media for 12 h. At 12 h after aphidicolin removal, all of the cells reached to G2/M transition (0 h) and at that time a set of plates were treated with JNK inhibitor SP600125 at 25 and 50 μM concentrations and collected after 4, 8 and 12 h. For flow cytometer analysis, cells were trypsinized, washed and fixed with 70% ethanol overnight at 4 °C at indicated time points. Washed cells were treated with 50 μg/mL RNase-A and then stained with 50 μg/mL propidium iodide. Flow cytometry analyses were performed using FACS Calibur machine and the FlowJo program. Western blot protocol described before was followed for detection of cell cycle-specific proteins.

### Hormone assays

After treatment with JNK inhibitors, supernatants were collected and stored in −30 °C until hormone assays were performed. Electrochemiluminescence immunoassay (ECLIA) kits specific for estradiol (Elecsys® Estradiol II, Cobas) and ACTIVE® Mullerian Inhibiting Substance/Anti-Mullerian Hormone (MIS/AMH) enzyme-linked immunosorbent (ELISA) (Diagnostic Systems Laboratories, Inc.) for AMH were used according to the manufacturer’s instructions to measure the corresponding hormone levels in the culture media. The analytical sensitivity of AMH and estradiol assays was 0.006 ng/mL and 5,0 pg/mL, respectively. All analyses were performed on Cobas® 6000 analyzer series (Roche Diagnostics, USA).

### Statistical analysis

Hormone levels and cell index readouts of xCELLigence system are continuous data and expressed as the mean + SD. Analysis of variance and multiple comparison post hoc test were applied to compare the data among the groups. Statistical analyses were done using SPSS for windows 20.0 statistical package program. The percentages of viable and apoptotic cells were compared between the groups using Fisher’ exact test. A *P*-value of < 0.05 was considered significant for all statistical tests.

## Electronic supplementary material

SUPPLEMENTARY FIGURE LEGENDS(DOCX 16 kb)

Supplementary Figure 1(TIF 1060 kb)

Supplementary Figure 2(TIF 578 kb)

Supplementary Figure 3(TIF 376 kb)

Supplementary Figure 4(TIF 374 kb)

Supplementary Figure 5(TIF 2367 kb)

Supplementary Figure 6(TIF 281 kb)

Supplementary Figure 7(TIF 177 kb)

## References

[1] Schumer ST, Cannistra SA (2003). Granulosa cell tumor of the ovary. J. Clin. Oncol..

[2] Young RH, Dickersin GR, Scully RE (1984). Juvenile granulosa-cell tumor of the ovary - a clinicopathological analysis of 125 cases. Am. J. Surg. Pathol..

[3] Shah SP (2009). Mutation of FOXL2 in granulosa-cell tumors of the ovary. N. Engl. J. Med..

[4] Deshpande DA (2014). Exploiting functional domains of GRK2/3 to alter the competitive balance of pro- and anticontractile signaling in airway smooth muscle. FASEB J..

[5] Nonis D, McTavish KJ, Shimasaki S (2013). Essential but differential role of FOXL2wt and FOXL2C134W in GDF-9 stimulation of follistatin transcription in co-operation with Smad3 in the human granulosa cell line COV434. Mol. Cell. Endocrinol..

[6] Anttonen M (2014). FOXL2, GATA4, and SMAD3 co-operatively modulate gene expression, cell viability and apoptosis in ovarian granulosa cell tumor cells. PLoS One.

[7] Vassal G (1988). Juvenile granulosa cell tumor of the ovary in children: a clinical study of 15 cases. J. Clin. Oncol..

[8] Wu H (2017). Juvenile granulosa cell tumor of the ovary: a clinicopathologic study. J. Pediatr. Adolesc. Gynecol..

[9] Auguste A (2015). Molecular analyses of juvenile granulosa cell tumors bearing AKT1 mutations provide insights into tumor biology and therapeutic leads. Hum. Mol. Genet..

[10] Kalfa N (2007). Extinction of FOXL2 expression in aggressive ovarian granulosa cell tumors in children. Fertil. Steril..

[11] Kalfa N (2006). Activating mutations of the stimulatory g protein in juvenile ovarian granulosa cell tumors: a new prognostic factor?. J. Clin. Endocrinol. Metab..

[12] Kaye SB, Davies E (1986). Cyclophosphamide, adriamycin, and cisplatinum for the treatment of advanced granulosa-cell tumor, using serum estradiol as a tumor-marker. Gynecol. Oncol..

[13] Rey RA (1996). Antimullerian hormone as a serum marker of granulosa cell tumors of the ovary: Comparative study with serum alpha-inhibin and estradiol. Am. J. Obstet. Gynecol..

[14] Johnson GL, Lapadat R (2002). Mitogen-activated protein kinase pathways mediated by ERK, JNK, and p38 protein kinases. Science.

[15] Weston CR, Davis RJ (2007). The JNK signal transduction pathway. Curr. Opin. Cell Biol..

[16] Johnson RS, Vanlingen B, Papaioannou VE, Spiegelman BM (1993). A null mutation at the c-Jun locus causes embryonic lethality and retarded cell-growth in culture. Genes Dev..

[17] Oktem O, Buyuk E, Oktay K (2011). Preantral follicle growth is regulated by c-Jun-N-terminal kinase (JNK) pathway. Reprod. Sci..

[18] Oktem O, Oktay K (2008). Follicle stimulating hormone regulates granulosa cell mitosis through c-Jun n terminal kinase (JNK) pathway. Reprod. Sci..

[19] Stein LS, Stoica G, Tilley R, Burghardt RC (1991). Rat ovarian granulosa cell culture: a model system for the study of cell-cell communication during multistep transformation. Cancer Res..

[20] Yuksel A (2015). The magnitude of gonadotoxicity of chemotherapy drugs on ovarian follicles and granulosa cells varies depending upon the category of the drugs and the type of granulosa cells. Hum. Reprod..

[21] Mingo-Sion AM, Marietta PM, Koller E, Wolf DM, Van Den Berg CL (2004). Inhibition of JNK reduces G2/M transit independent of p53, leading to endoreduplication, decreased proliferation, and apoptosis in breast cancer cells. Oncogene.

[22] Miyamoto-Yamasaki Y, Yamasaki M, Tachibana H, Yamada K (2007). Induction of endoreduplication by a JNK inhibitor SP600125 in human lung carcinoma A 549 cells. Cell Biol. Int..

[23] Mili D, Abid K, Rjiba I, Kenani A (2016). Effect of SP600125 on the mitotic spindle in HeLa cells, leading to mitotic arrest, endoreduplication and apoptosis. Mol. Cytogenet..

[24] Grassi ES (2015). SP600125 has a remarkable anticancer potential against undifferentiated thyroid cancer through selective action on ROCK and p53 pathways. Oncotarget.

[25] Oktem O, Urman B (2010). Understanding follicle growth in vivo. Hum. Reprod..

[26] Moumne L (2008). The mutations and potential targets of the forkhead transcription factor FOXL2. Mol. Cell Endocrinol..

[27] Crisponi L (2001). The putative forkhead transcription factor FOXL2 is mutated in blepharophimosis/ptosis/epicanthus inversus syndrome. Nat. Genet..

[28] Schmidt D (2004). The murine winged-helix transcription factor Foxl2 is required for granulosa cell differentiation and ovary maintenance. Development.

[29] Kobel M, Gilks CB, Huntsman DG (2009). Adult-type granulosa cell tumors and FOXL2 mutation. Cancer Res..

[30] Benayoun BA (2011). Transcription factor FOXL2 protects granulosa cells from stress and delays cell cycle: role of its regulation by the SIRT1 deacetylase. Hum. Mol. Genet..

[31] Fleming NI (2010). Aromatase is a direct target of FOXL2: C134W in granulosa cell tumors via a single highly conserved binding site in the ovarian specific promoter. PLoS One.

[32] L’Hote D (2012). Discovery of novel protein partners of the transcription factor FOXL2 provides insights into its physiopathological roles. Hum. Mol. Genet..

[33] Kim JH (2011). Differential apoptotic activities of wild-type FOXL2 and the adult-type granulosa cell tumor-associated mutant FOXL2 (C134W). Oncogene.

[34] Liu Z (2015). FOXO1/3 and PTEN depletion in granulosa cells promotes ovarian granulosa cell tumor development. Mol. Endocrinol..

[35] Richards JS (2012). Either Kras activation or Pten loss similarly enhance the dominant-stable CTNNB1-induced genetic program to promote granulosa cell tumor development in the ovary and testis. Oncogene.

[36] Kinross KM (2012). An activating Pik3ca mutation coupled with Pten loss is sufficient to initiate ovarian tumorigenesis in mice. J. Clin. Invest..

[37] Bittinger S, Alexiadis M, Fuller PJ (2009). Expression status and mutational analysis of the PTEN and P13K subunit genes in ovarian granulosa cell tumors. Int. J. Gynecol. Cancer.

[38] Rico C (2012). Pharmacological targeting of mammalian target of rapamycin inhibits ovarian granulosa cell tumor growth. Carcinogenesis.

[39] Zhang H (2000). Characterization of an immortalized human granulosa cell line (COV434). Mol. Hum. Reprod..

[40] Nishi Y (2001). Establishment and characterization of a steroidogenic human granulosa-like tumor cell line, KGN, that expresses functional follicle-stimulating hormone receptor. Endocrinology.

[41] Bayasula (2012). Establishment of a human nonluteinized granulosa cell line that transitions from the gonadotropin-independent to the gonadotropin-dependent status. Endocrinology.

[42] Oktem O, Senbabaoglu F, Muftuoglu M, Urman B (2013). Real-time analysis of the growth of human granulosa cells using an impedance-based signal processing system: a new technology for translational research in human reproduction. Hum. Reprod..

[43] Oktem O, Buyuk E, Oktay K (2011). Preantral follicle growth is regulated by c-Jun-N-terminal kinase (JNK) pathway. Reprod. Sci..

