# Supplementary material for: Endogenous c-Jun N-terminal kinase (JNK) activity marks the boundary between normal and malignant granulosa cells
Source: Cell Death Dis. 2018 Mar 16;9(4):421. doi: 10.1038/s41419-018-0459-3 (PMC5856777; doi:10.1038/s41419-018-0459-3)
Supplement: Supplementary file 1 — SUPPLEMENTARY FIGURE LEGENDS(DOCX 16 kb) [file 41419_2018_459_MOESM1_ESM.docx]

**SUPPLEMENTARY FIGURE LEGENDS**

**Supplementary Fig 1:** Treatment with JNK inhibitor SP600125 (25 µM) for 30 min and transfection with JNK siRNA (50 nM) successfully inhibited the phosphorylation of c-Jun^Ser63^ in KGN cells (Scale bar: 100 µm).

**Supplementary Fig 2:** Inhibition of JNK pathway via pharmacological JNK inhibitors or JNK siRNA blocked the proliferation of KGN cells. Real-time growth curves obtained from the xCELLigence platform demonstrate that there is a dose-dependent decrease in in vitro proliferation of the cells after JNK inhibition (2A). Mean cell index as a measure of viable cell mass at the end of experiment period confirmed reduced proliferation of the cells after JNK inhibition either pharmacologically or via siRNA (shown as graph bar in 2B). Additionally, reduced proliferation was associated with decreased production of estradiol and AMH in these cells (2C).

**Supplementary Fig 3:** JNK inhibition in asynchronous KGN cells is correlated with a reduction in mitotic activity as verified by lowered expression of mitosis marker phospho-histone H3^Ser10^ on immunofluorescence analysis (3A). While 14% of the control cells were stained positive for phospho-histone H3^Ser10^, this ratio was decreased when JNK was inhibited either pharmacologically (2.5%) or via siRNA (3%) (3B) (Scale bar: 100 µm).

**Supplementary Fig 4:** JNK inhibition at G1/S is associated with decreased DNA synthesis in GCT cell line KGN. There was a significant reduction in EdU uptake of the cells after JNK inhibition with pharmacological inhibitor (6%) or siRNA (9%) compared to control cells (37%, p<0.01) (4A and 4B) (Scale bar: 100 µm).

**Supplementary Fig 5:** High-magnification images for the immunofluorescence staining experiments in C0V 434 cell line for phospho-c-Jun and phospho-histone-H3 (Scale bar: 50 µm).

**Supplementary Fig 6:** Comparison of endogenous JNK activity among different types of neoplastic and non-neoplastic human granulosa cells with kinase assay. JNK activity is significantly higher in GCT samples of adult and juvenile types as well as GCT cell lines COV434 and KGN in comparison to non-neoplastic primary human luteinized granulosa cells (HLGCs) obtained from three different IVF patients. There is increased expression of phospho-c-Jun at Ser63 and Ser73 residues in GCT samples and cell lines in comparison to non-neoplastic primary granulosa cells (shown as western blot image in 6A and graphic bars in 6B after quantification of the intensity of the signals).

**Supplementary Fig 7:** Ex vivo treatment of the adult type GCT fresh tumor samples with 3 different doses of JNK inhibitor AS601245 for 24 hours caused a dose-dependent decrease in their estradiol and AMH productions.
