# Supplementary figures and images for: Endogenous c-Jun N-terminal kinase (JNK) activity marks the boundary between normal and malignant granulosa cells
Source: Cell Death Dis. 2018 Mar 16;9(4):421. doi: 10.1038/s41419-018-0459-3 (PMC5856777; doi:10.1038/s41419-018-0459-3)

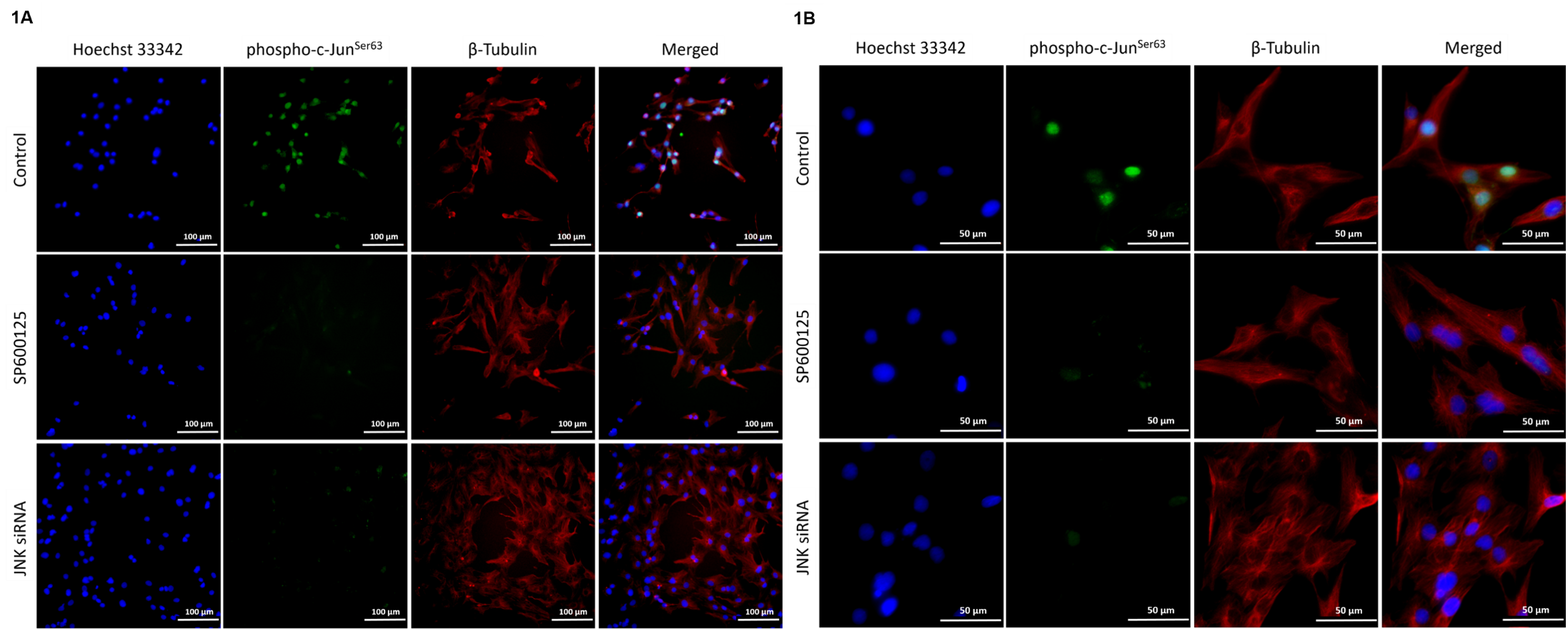

Supplement: Supplementary file 2 — Supplementary Figure 1(TIF 1060 kb) [file 41419_2018_459_MOESM2_ESM.tif]

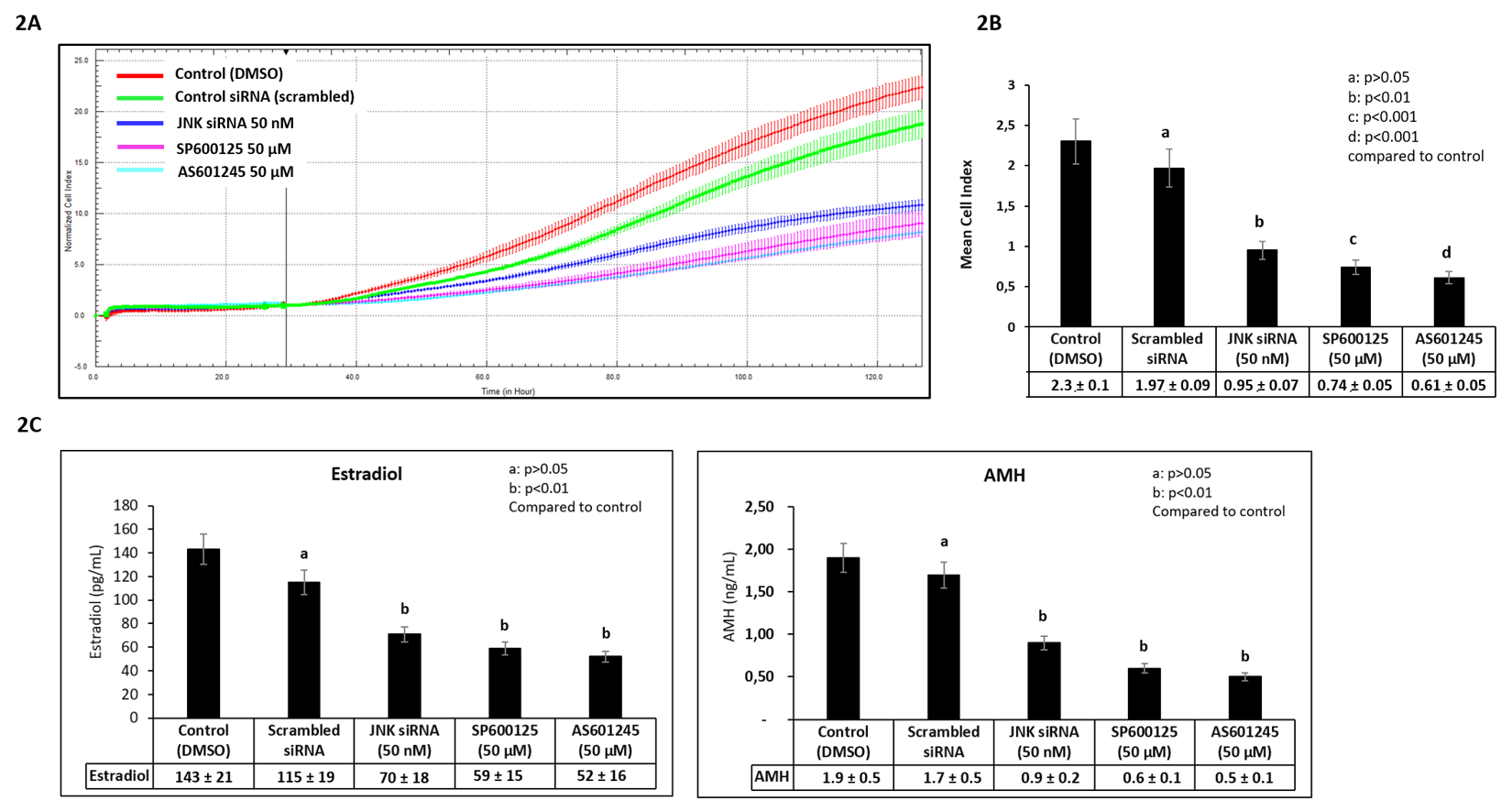

Supplement: Supplementary file 3 — Supplementary Figure 2(TIF 578 kb) [file 41419_2018_459_MOESM3_ESM.tif]

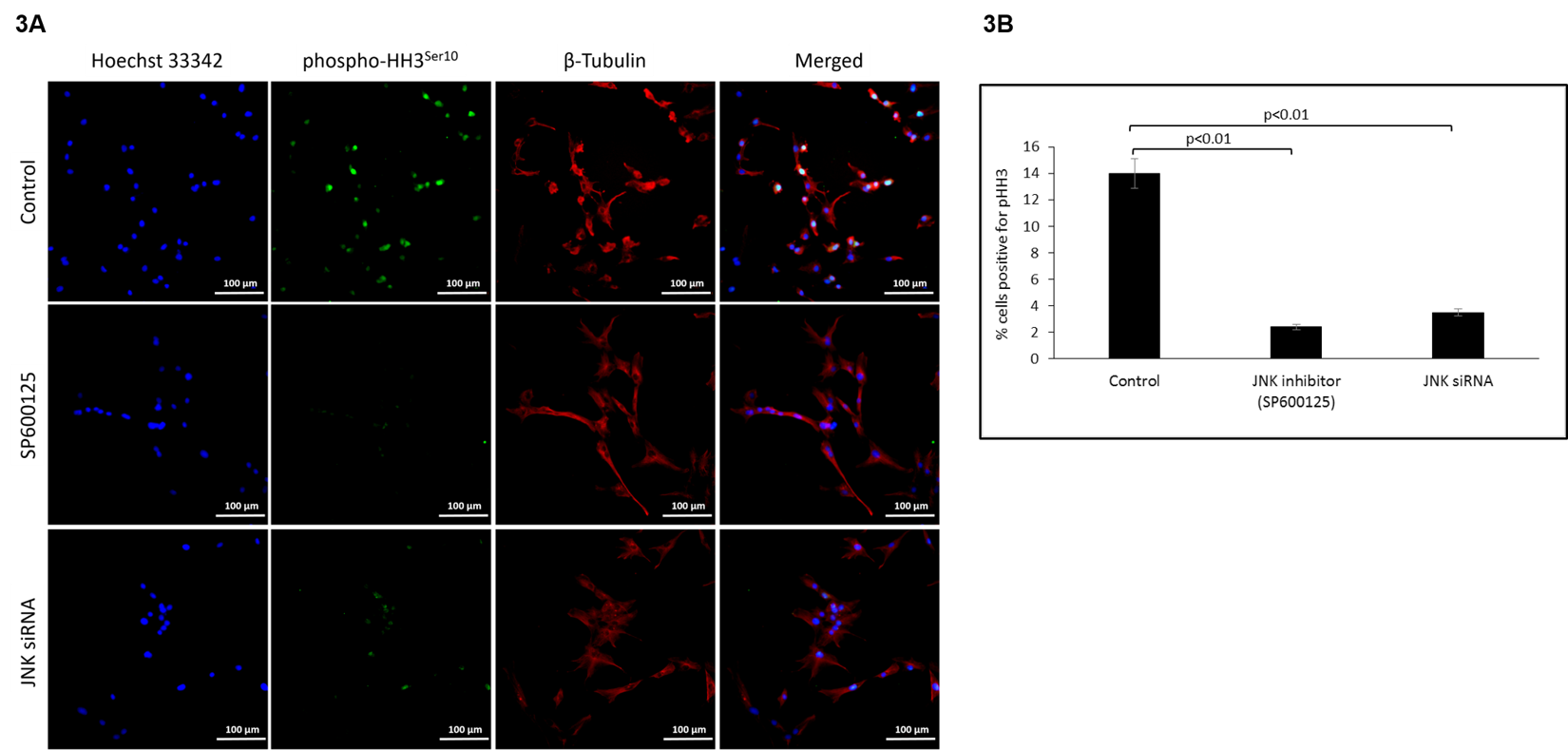

Supplement: Supplementary file 4 — Supplementary Figure 3(TIF 376 kb) [file 41419_2018_459_MOESM4_ESM.tif]

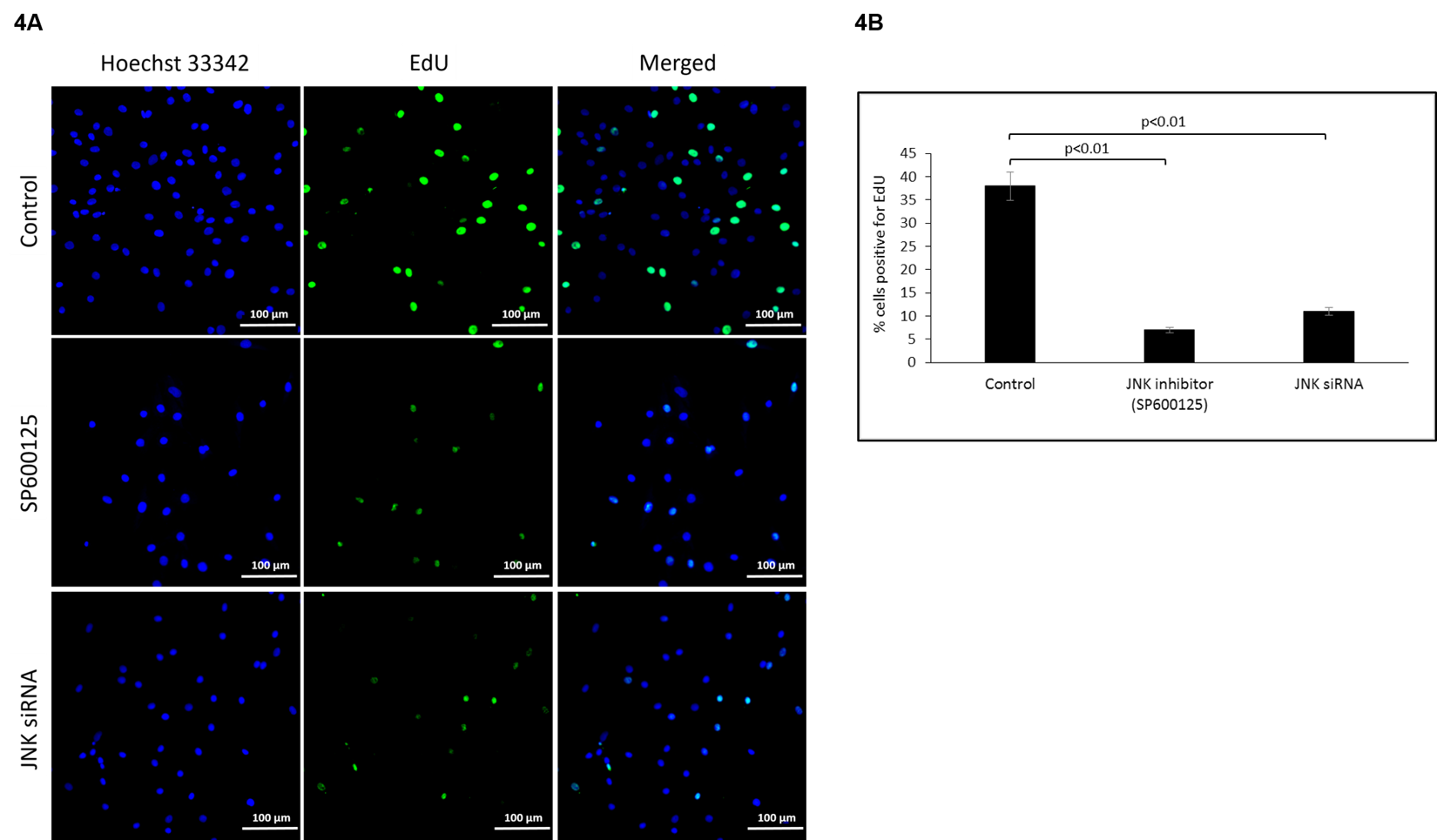

Supplement: Supplementary file 5 — Supplementary Figure 4(TIF 374 kb) [file 41419_2018_459_MOESM5_ESM.tif]

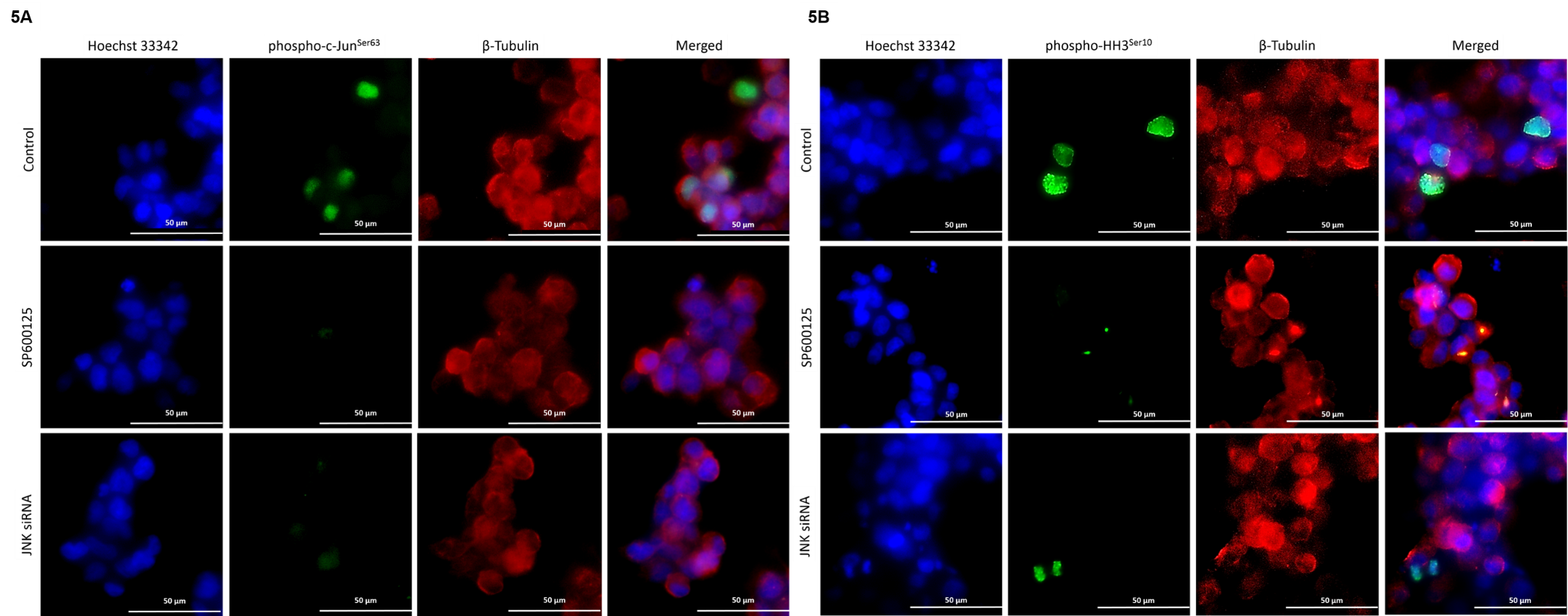

Supplement: Supplementary file 6 — Supplementary Figure 5(TIF 2367 kb) [file 41419_2018_459_MOESM6_ESM.tif]

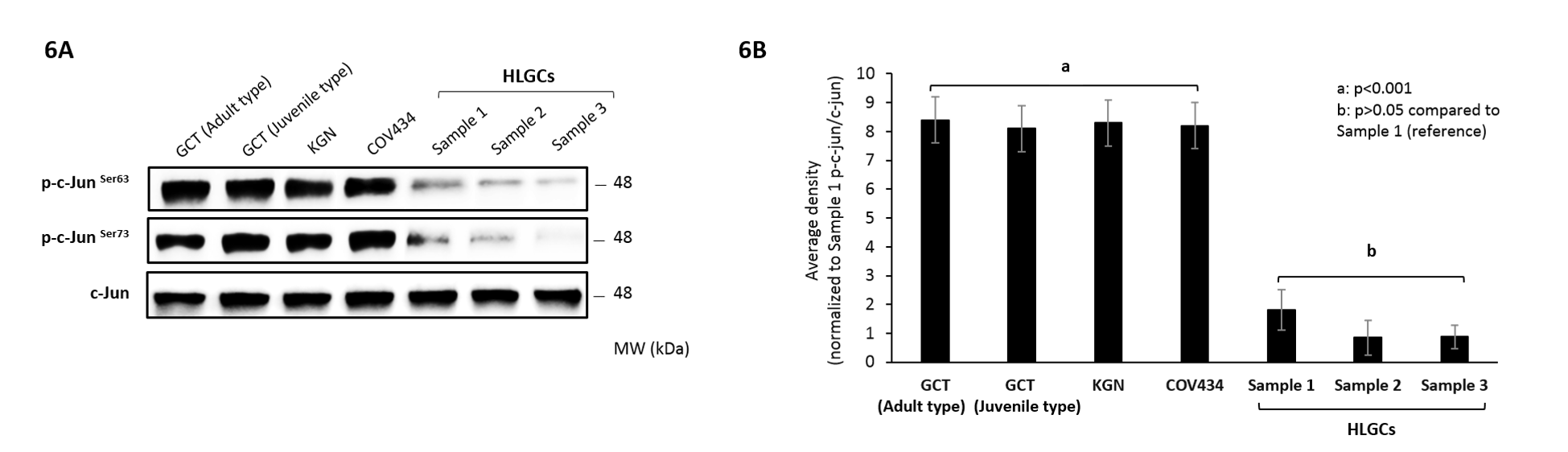

Supplement: Supplementary file 7 — Supplementary Figure 6(TIF 281 kb) [file 41419_2018_459_MOESM7_ESM.tif]

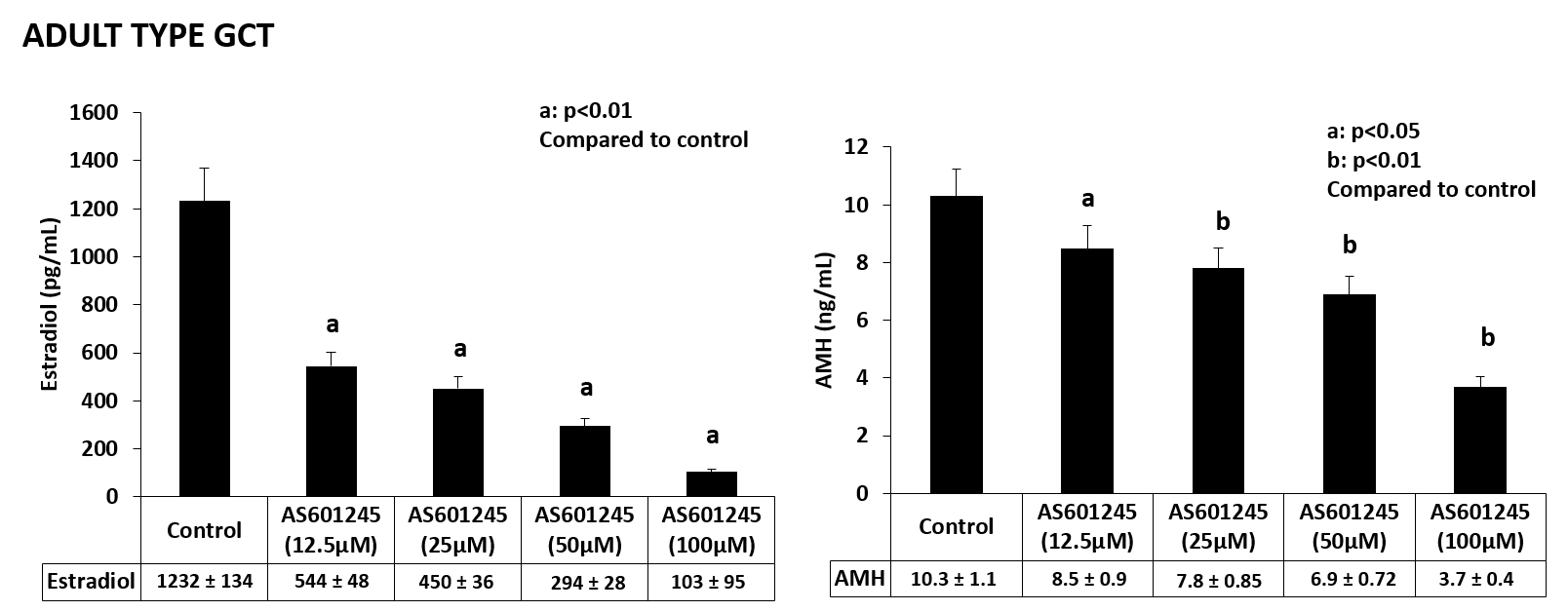

Supplement: Supplementary file 8 — Supplementary Figure 7(TIF 177 kb) [file 41419_2018_459_MOESM8_ESM.tif]
